# Parental psychological control and adolescents’ academic adjustment: a moderated serial mediation model

**DOI:** 10.3389/fpsyg.2026.1910535

**Published:** 2026-10-19

**Authors:** Shangjie Meng, Xingnan Zhan, Chengzhe Guo

**Affiliations:** 1 International College, Krirk University, Bangkok, Thailand; 2 Faculty of Education, Beijing Normal University, Beijing, China

**Keywords:** academic adjustment, academic buoyancy, growth mindset, learning engagement, parental psychological control

## Abstract

**Background:**

Parental psychological control has been associated with poorer academic adjustment among adolescents, yet the indirect associations and moderating factors involved remain insufficiently understood. Guided by self-determination theory, this study examined the association between parental psychological control and adolescents’ academic adjustment, the indirect associations involving academic buoyancy and learning engagement, and the moderating role of growth mindset.

**Methods:**

A total of 620 Chinese adolescents completed standardized questionnaires assessing parental psychological control, academic buoyancy, learning engagement, growth mindset, and academic adjustment. Data were analyzed using SPSS with the PROCESS macro to test a moderated serial mediation model.

**Results:**

Parental psychological control was negatively associated with adolescents’ academic adjustment (*p <* 0.001). Three significant statistical indirect associations were identified. The indirect association involving academic buoyancy accounted for 18.09% of the total indirect effect, and that involving learning engagement accounted for 22.94%. The indirect association involving academic buoyancy and learning engagement in the specified serial model accounted for 8.56% of the total indirect effect. Moderation analyses showed that the negative associations of parental psychological control with academic adjustment, academic buoyancy, and learning engagement were weaker at higher levels of growth mindset.

**Conclusion:**

The findings suggest that parental psychological control is associated with adolescents’ academic adjustment through statistical indirect associations involving academic buoyancy and learning engagement, with these associations varying across levels of growth mindset. The cross-sectional design precludes causal conclusions, and the proposed relationships require further examination using longitudinal and intervention designs.

## 1 Introduction

Adaptation refers to the psychological adjustment that individuals use to meet problems, challenges, and external demands (Bibi et al., 2018). In schooling, scholars describe this as academic adjustment (AA) (Safriani and Muhid, 2022). The present study focuses on investment in school as a specific aspect of AA, reflecting the importance adolescents attach to doing well academically (Cheung et al., 2013). As a key indicator of adolescents’ development in school, AA relates to knowledge acquisition and competence growth and is also associated with long-term mental health and social adaptation (Chen et al., 2024). Poor AA among adolescents is often associated with declines in academic achievement (Chen et al., 2024; Branje and Morris, 2021) and with greater risks of anxiety and depressive symptoms (Ershadi Chahardeh, 2020). Ongoing difficulties with AA may also be associated with difficulties in critical thinking and other competencies (Wang et al., 2024). Investigating the correlates of adolescents’ AA may therefore inform hypotheses for future research on educational support.

Prior work shows that parental psychological control (PPC) (Cui et al., 2014), low socioeconomic status (Kim et al., 2023), COVID-19-related disruptions (Branje and Morris, 2021), and cumulative risks tied to left-behind experiences (Wang et al., 2024) are negatively associated with adolescents’ AA. By contrast, higher academic buoyancy (AB) (Liu, 2024), stronger learning engagement (LE) (García-Ros et al., 2018), and effective emotion regulation are positively associated with better adjustment. Yet most studies emphasize direct associations from external or individual factors to AA. The potential serial mediation of AB and LE between PPC and AA remains insufficiently tested. Growth mindset (GM), as a positive psychological resource, may also moderate these associations, but empirical evidence is limited. These gaps limit an integrated understanding of the factors associated with adolescents’ AA.

Based on Self-Determination Theory (SDT), this study proposes a moderated mediation model linking PPC, AB, LE, GM, and AA. The theory states that satisfaction of autonomy, competence, and relatedness needs supports sustained engagement and organizes behavior. Within this conceptual model, PPC is expected to show a negative association with AA, with AB and LE representing a hypothesized serial indirect association alongside the direct association. We further expect GM to moderate the direct and indirect associations. By examining these indirect associations and their variation across levels of GM, this study seeks to extend research on adolescents’ AA and to identify hypotheses for future longitudinal and intervention studies.

## 2 Theoretical foundation and research hypotheses

### 2.1 Theoretical foundation

From the perspective of self-determination theory (Deci and Ryan, 2012), this study explores the association of PPC with adolescents’ AA, focusing on the sequential involvement of AB and LE as well as the moderating role of GM. SDT posits that fulfilling basic psychological needs is central to adaptive behavior and psychological wellbeing (Nagpaul and Chen, 2019). Researchers have widely applied this framework in educational psychology and adolescent development, such as family influences, learning motivation, and academic performance (Dean et al., 2021; Nogg et al., 2021; Nishimura and Sakurai, 2017; Shih, 2015). Within this framework, lower satisfaction of autonomy, competence, and relatedness needs provides a possible theoretical explanation for the association between PPC and AA. PPC is hypothesized to be associated with lower AB, and lower AB is expected to be associated with lower LE and poorer AA. These proposed associations motivate the specified ordering of AB and LE in the model, but the present cross-sectional data cannot establish this temporal sequence or test need satisfaction as an explanatory mechanism. Prior findings on these associations remain mixed, especially for the serial mediation of AB and LE.

Building on SDT, the present research formulates a conditional process model that integrates statistical mediation and moderation. The model examines indirect associations between PPC and AA involving AB and LE, and whether these associations vary across levels of GM. This approach may inform hypotheses about family context and AA for subsequent longitudinal and intervention research.

### 2.2 Parental psychological control and adolescents’ academic adjustment

PPC refers to the use of psychological tactics to regulate children’s behavior (Scharf and Goldner, 2018). Studies have shown that higher PPC is associated with poorer AA among adolescents (Lerner et al., 2022; Cui et al., 2014). Adolescence is a period marked by movement toward autonomy and a desire for respect as independent individuals (Scharf and Goldner, 2018). Strategies such as guilt induction, withdrawal of affection, and overinvolvement may be associated with lower satisfaction of basic psychological needs (Xu et al., 2024). Learning to appease parents, avoid anxiety, or resist control, rather than to express interest or personal goals, may be associated with lower intrinsic motivation and autonomy (Lerner et al., 2022), as well as poorer AA. Negative parental messages may also be associated with self-doubt and feelings of helplessness when facing academic difficulties, alongside lower resilience and confidence (Xu et al., 2024). For example, Cui et al. (2014) reported a negative correlation between PPC and adolescents’ adaptation. Accordingly, the present research puts forward the following hypotheses:

*H1*: PPC is negatively associated with adolescents’ AA.

### 2.3 The mediating role of academic buoyancy

AB is defined by researchers as a learner’s resilience in the face of typical academic pressures and minor adversities inherent in the school environment (Martin and Marsh, 2008). Evidence from multiple studies shows that AB is positively associated with AA (Liu, 2024; Kritikou and Giovazolias, 2022; Safriani and Muhid, 2022). Buoyant students tend to report positive emotions, sustained learning motivation, and recovery from temporary difficulties, alongside less disengagement or avoidance (Wang et al., 2025). AB may also be associated with viewing everyday academic challenges as manageable. Greater AB and LE are associated with stronger academic performance, a greater sense of school belonging, and better overall adjustment (Liu, 2024). For example, Safriani and Muhid (2022) studied high school population throughout the COVID-19 pandemic and found that AB was positively associated with AA.

Currently, no study has explicitly explored the relationship between PPC and AB. Guided by SDT, we propose that PPC may be associated with lower satisfaction of students’ autonomy, competence, and relatedness needs, which provides a possible explanation for its hypothesized association with AB (Nagpaul and Chen, 2019). Adolescents who perceive greater parental control may also report more threatening appraisals of daily challenges and fewer cognitive and emotional resources. In contrast, parental democratic involvement may be associated with greater satisfaction of basic psychological needs and higher AB (Chen and Mok, 2023). These theoretical propositions suggest an association between PPC and AB, but do not establish changes in AB over time. Earlier work has explored the mediating effect of AB on the connection between academic self-efficacy and scholastic achievement in secondary school samples (Lei et al., 2022) and between perceived social support and LE in university samples (Javadi-Elmi et al., 2022). However, whether AB mediates the association between PPC and AA remains uncertain. This research proposes that higher PPC is associated with lower AB, and that lower AB is associated with poorer AA. Accordingly, this study includes AB in the model to test its mediating role between PPC and AA. Therefore, this paper advances the following hypotheses:

*H2*: AB mediates the association between PPC and adolescents’ AA.

### 2.4 The mediating role of learning engagement

Astin (1985) defined LE as the expenditure of physical and psychological energy. It is context specific and includes quantitative and qualitative components. Prior research shows that LE is positively associated with AA (Wang et al., 2021; García-Ros et al., 2018). Students who report higher engagement participate more in class, devote sustained effort to tasks, and persist when difficulties arise. These behaviors are associated with better cognitive, emotional, and behavioral adaptation to school demands (Wang et al., 2021). Adolescents with strong engagement also report focused cognition and proactive behaviors, which are associated with adaptation to academic requirements and the management of learning challenges (An et al., 2022). For example, García-Ros et al. (2018) examined adolescents aged 12–18 and reported a positive association between engagement and AA. Based on this evidence, this study posits that adolescents with greater LE also show better AA, suggesting that active engagement is associated with stronger overall adaptability.

Studies also report a negative association between PPC and LE (Zhang and Peng, 2025). Higher levels of PPC are often linked to lower LE among adolescents. Guided by SDT, PPC may be associated with lower perceived autonomy, competence, and relatedness with parents (Xu et al., 2024). Lower need satisfaction provides a possible theoretical explanation for the association between PPC and LE. Greater psychological control may also be associated with viewing learning as an imposed obligation, emotional exhaustion, cognitive avoidance, and lower engagement (Zhang and Peng, 2025). Conversely, lower PPC may be associated with greater need satisfaction and higher engagement. For instance, Zhang and Peng (2025) reported a negative correlation between PPC and children’s LE among youths with lower social mobility. Earlier studies have reported statistical mediation involving LE in the association between achievement goal orientation and AA in first-year students (Wang et al., 2021) and between social support and academic achievement (Zhang and Qian, 2024). It’s role in the association between PPC and AA remains unclear. This study therefore includes LE as a mediator to examine the indirect association between PPC and AA involving LE. These considerations lead to the following research hypotheses:

*H3*: LE mediates the relationship between PPC and adolescents’ AA.

### 2.5 The serial mediating role of academic buoyancy and learning engagement

Prior research has consistently documented a significant positive link between AB and LE (Ren et al., 2025; Zhao et al., 2025; Ershadi Chahardeh, 2020). Adolescence represents a critical phase for fostering psychological resilience. AB concerns how students manage everyday academic setbacks, such as disappointing exam performance and routine study stress (Azemi et al., 2021). Greater AB may be associated with sustained psychological energy, concentration, and active participation in classroom interaction and self-directed study (Ren et al., 2025). High AB also relates to timely strategy adjustment and continued behavioral engagement when difficulties occur (Ershadi Chahardeh, 2020). For example, Azemi et al. (2021) found a significant positive correlation between AB and LE among middle school students. Ershadi Chahardeh (2020) likewise reported that stronger buoyancy is associated with higher engagement among high school students. However, studies have not systematically tested whether AB and LE operate as serial mediators between PPC and adolescents’ AA. This study therefore proposes an integrated model to examine the statistical indirect association between PPC and adolescents’ AA involving AB and LE in a theoretically specified order. The study therefore formulates this hypothesis:

*H4*: AB and LE serve as serial mediators between PPC and adolescents’ AA.

### 2.6 The moderating role of growth mindset

GM is that intelligence and abilities are not fixed but can be cultivated through dedication, learning, and hands-on practice (Dweck, 2006). Scholars have reported a positive association between GM and students’ AA (Wang et al., 2024). When adolescents encounter academic setbacks or external pressure, those with a GM see obstacles not as attacks on their self-esteem, but as chances to develop mastery (Walker and Jiang, 2022). This appraisal may be associated with greater perceived psychological resources and better AA. Wang et al. (2024) examined left-behind middle school students and found that GM moderated the association between cumulative risk and AA. However, researchers have not yet tested whether GM moderates the association between PPC and adolescents’ AA. This study proposes that the negative association between PPC and AA is weaker among adolescents with higher GM. One possible explanation is that these adolescents appraise PPC as a manageable challenge rather than a denial of self-worth, and that such appraisals are associated with higher AB and LE. Strategy adjustment and persistence may also be relevant to these associations, although these explanations are not directly tested in this study. Building upon this rationale, the following hypotheses are put forward:

*H5*: GM moderates the association between PPC and adolescents’ AA.

*H6*: GM moderates the association between PPC and adolescents’ AB.

*H7*: GM moderates the association between PPC and adolescents’ LE.

The conceptual model corresponding to these hypotheses is shown below.

### 2.7 Hypothesized model

Figure 1 summarizes the hypothesized associations among the study variables. H1 proposes a negative overall association between PPC and AA. Three specific indirect associations are hypothesized within the serial mediation model: through AB (H2), through LE (H3), and through AB and LE in sequence (H4). GM is expected to moderate the direct association between PPC and AA after accounting for AB and LE (H5), the association between PPC and AB (H6), and the association between PPC and LE after accounting for AB (H7). These associations are expected to be less negative at higher levels of GM. The ordering of AB and LE reflects the theoretical specification of the model and does not establish temporal precedence or causality in this cross-sectional study.

**Figure 1:**
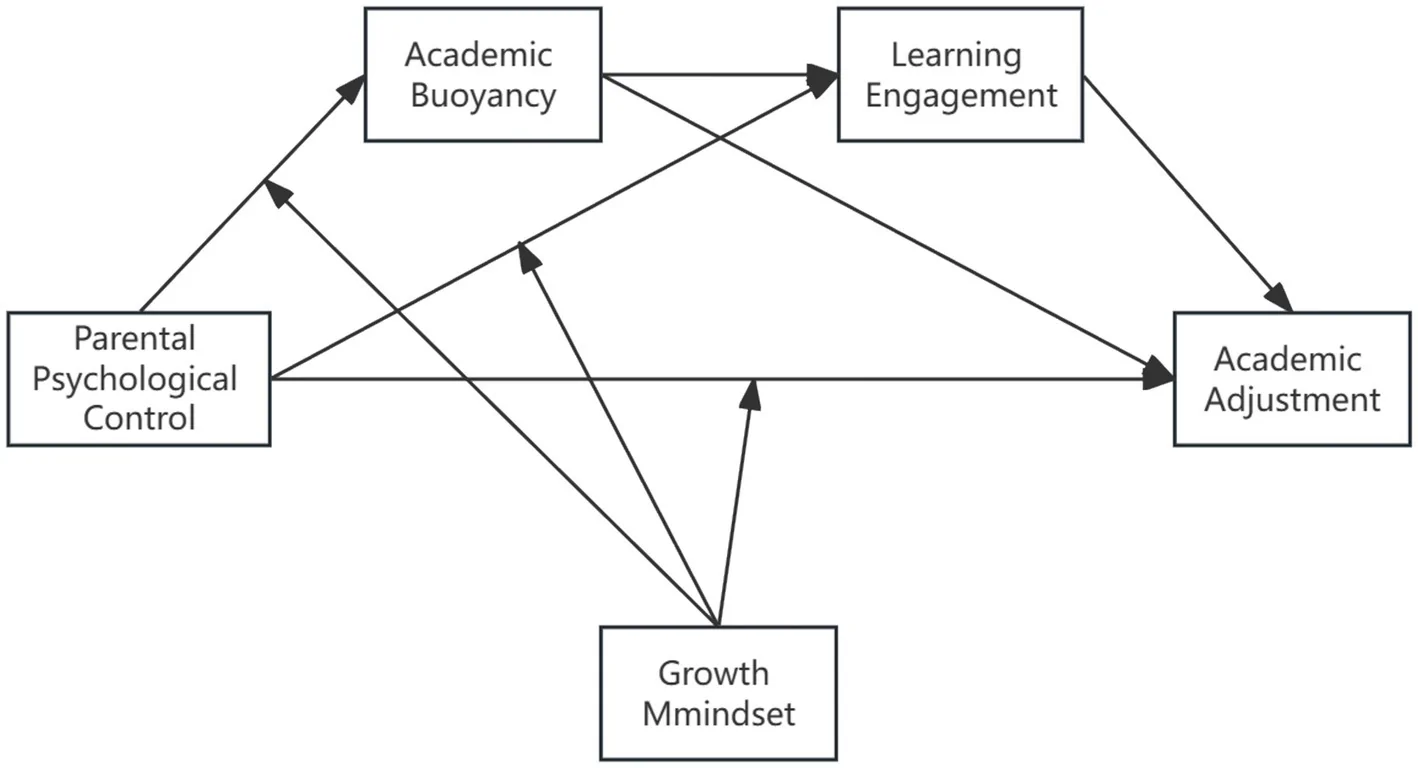
Conceptual model of the study.

## 3 Methods

### 3.1 Sample and data collection

The Ethics Committee of Beijing Normal University approved this study (Approved No. BNU-2025-06-049). Participants were students in Grades 7–12 recruited from multiple junior and senior high schools in China using convenience sampling. All stages of the study, including participant enrollment, data collection, and data analysis, were conducted in accordance with the ethical standards of the Declaration of Helsinki. The questionnaire was distributed via Wenjuanxing, an online survey platform, from August to September 2025, with assistance from teachers at the participating schools. Before participation, students and their parents or legal guardians were informed about the research objectives and procedures. Informed consent was obtained from all parents or legal guardians, and assent was obtained from all student participants; both were obtained through online confirmation. Participation was entirely voluntary. Although the use of convenience sampling could lead to self-selection bias and restrict broader applicability, this method was considered the most suitable given the current practical and resource limitations.

Following Kline (2018)‘s recommendation of at least 10 respondents per item, the 54-item questionnaire required a minimum of 540 cases. After incorporating a 20% buffer for expected participant attrition, the final target sample size was set at 648 (54 × 10 × 1.20). A total of 700 questionnaires were issued, and 651 valid returns were obtained initially. During the data-screening process, 31 cases were discarded due to either substantial item nonresponse exceeding 20% or a pronounced pattern of extreme responding, defined as more than 80% of answers falling at the scale extremes. Such patterns can produce serious bias, including floor or ceiling effects, and reduce analytic accuracy (Wang et al., 2012). The final analytic sample comprised 620valid responses.

Table 1 reports the demographics. The sample included 277 males (44.7%) and 343 females (55.3%). A total of 130 respondents (21.0%) were in the second year of junior high school. Regarding fathers’ education, 35.5% (*n* = 220) had completed high school, technical, or vocational schooling; for mothers, 41.6% (*n* = 258) had completed junior high school. Concerning residence, 29.7% (*n* = 184) lived in large cities (provincial capitals or municipalities). For self-reported academic performance, 37.6% (*n* = 233) indicated a “good” level. AA differed significantly only by gender (*p* < 0.05).

**Table 1:** Sample demographic characteristics.

Variables	Options	*N*	Percentage (%)	*t/F* - AA
Gender	Male	277	44.7	2.340*
	Female	343	55.3	
Grade	Grade 7	62	10.0	0.529
	Grade 8	130	21.0	
	Grade 9	129	20.8	
	Grade 10	97	15.6	
	Grade 11	122	19.7	
	Grade 12	80	12.9	
Father’s educational level	Elementary school or below	95	15.3	0.805
	Junior high school	178	28.7	
	High school/vocational secondary school/technical school	220	35.5	
	Associate degree/bachelor’s degree	101	16.3	
	Master’s degree or above	26	4.2	
Mother’s educational level	Elementary school or below	113	18.2	0.777
	Junior high school	258	41.6	
	High school/vocational secondary school/Technical school	126	20.3	
	Associate degree/bachelor’s degree	87	14.0	
	Master’s degree or above	36	5.8	
Family residence location	Major cities (provincial capitals, municipalities)	184	29.7	0.361
	Small-medium cities (prefecture-level cities, county-level cities)	184	29.7	
	Towns	170	27.4	
	Rural areas	82	13.2	
Relative academic performance in class	Excellent (top 10%)	106	17.1	0.900
	Good (top 11–30%)	233	37.6	
	Satisfactory (31–70%)	152	24.5	
	Needs improvement (bottom 30%)	129	20.8	

*p < 0.05. AA, academic adjustment.

### 3.2 Measurement instruments

#### 3.2.1 Parental Psychological Control Scale (PPCS)

This study measured adolescents’ perceived PPC using the Chinese adaptation of Barber (1996) PPCS, translated and revised by Wang et al. (2007). The scale consists of three dimensions: authoritarian assertion, guilt induction, and love withdrawal. It includes 18 items (e.g., “My parents tell me all the things they have done for me”), scored on a 5-point Likert scale from 1 (strongly disagree) to 5 (strongly agree). All items were positively keyed, with higher aggregate scores indicating stronger perceived PPC. Previous research has supported the scale’s reliability and validity among Chinese high school students (Xu et al., 2020). In the present study, the instrument showed excellent internal consistency (Cronbach’s *α* = 0.921).

#### 3.2.2 Academic Buoyancy Scale (ABS)

AB was assessed using the ABS developed by Martin and Marsh (2008), which evaluates students’ capacity to manage everyday academic difficulties and setbacks. The construct of AB was measured with four positively framed items, such as “I don’t let academic pressure get me down.” Each item was scored on a 5-point Likert continuum from 1 (strongly disagree) to 5 (strongly agree), with larger totals indicating stronger AB. The scale has previously shown adequate psychometric quality in samples of Chinese junior high school students (Chen et al., 2024). In the present sample, reliability was acceptable (Cronbach’s *α* = 0.881).

#### 3.2.3 Utrecht Work Engagement Scale-Student-9 (UWES-S-9)

LE was assessed by the Chinese version of the UWES-S-9 developed by Schaufeli et al. (2006). The scale reflects a positive and fulfilling study-related state with three facets: vigor, dedication, and absorption. It contains 9 items (e.g., “I feel bursting with energy when studying”). Items 1, 2, and 5 represent vigor, 3, 4, and 7 represent dedication, and 6, 8, and 9 represent absorption. Responses were recorded on a seven-point Likert scale (1 = never, 7 = always/every day), with higher scores indicating greater LE. Previous research involving Chinese students has demonstrated strong reliability and validity (Wei et al., 2025). In this sample, the measure showed high internal consistency (Cronbach’s *α* = 0.945).

#### 3.2.4 Implicit Theories of Intelligence Scale (ITIS)

Dweck (2013) ITIS to evaluate GM about the malleability of intelligence. The instrument includes 8 items (e.g., “My intelligence is fixed and cannot be changed much through effort”) across two dimensions: Items assessing GM were coded in the positive direction, whereas those reflecting a fixed mindset were reverse-coded. Responses were rated on a 6-point Likert scale from 1 (strongly agree) to 6 (strongly disagree), with higher scores indicating stronger GM. The scale has shown good psychometric properties among Chinese students (Kwan et al., 2022). In this study, Cronbach’s α was 0.888.

#### 3.2.5 Investment in School Scale (ISS)

AA was operationalized in terms of investment in school, using the eight-item measure adapted from Pomerantz et al. (2000) and used by Cheung et al. (2013). Previous research involving Chinese adolescents has treated investment in school as one aspect of academic adjustment (Cheung et al., 2013). The measure was therefore selected to capture adolescents’ valuing of academic success, rather than the full range of academic adjustment. In the Chinese context these are language arts, mathematics, biology, and English. For each subject, adolescents rated the importance of achieving good grades (e.g., “How important is it for you to earn high grades in mathematics?”) and the importance of avoiding poor performance (e.g., “How important is it for you to avoid performing poorly in language arts?”). The eight items, all positively scaled from 1 (not at all important) to 7 (very important), were averaged. Higher scores indicate greater investment in school, the specific aspect of AA assessed in this study. Reported internal consistency ranges from 0.91 to 0.94 in United States samples and from 0.88 to 0.91 in Chinese samples (Cheung et al., 2013). The data demonstrated high internal consistency (Cronbach’s *α* = 0.906).

A standardized cross-cultural translation procedure to adapt the original scales in this study. A bilingual psychologist and a linguist produced independent forward translations. A separate team of psychology and language specialists who had not seen the originals conducted back translation. The research team reconciled discrepancies through iterative review to ensure semantic equivalence. A pilot with 20 Chinese secondary school students informed revisions concerning clarity, cultural fit, and meaning consistency. The finalized Chinese versions met psychometric requirements.

### 3.3 Data analysis

This study used SPSS 27.0 for all analyses. Normality was evaluated using Kim (2013) criteria, where absolute skewness < 2 and absolute kurtosis < 7 indicated approximate normality. The main variables (PPC, AB, LE, AA, and GM) met this criterion. Descriptive statistics included means (M) and standard deviations (SD). Pearson correlations were computed to examine associations among variables. The hypotheses were examined using the PROCESS macro (Hayes, 2017). PPC was specified as the predictor, AA as the outcome, AB as the first mediator, and LE as the second mediator. Model 6 estimated the direct association and the three specific indirect associations corresponding to H2–H4. Model 85 extended this specification by including GM as a moderator of the direct association between PPC and AA after accounting for AB and LE (H5), and the associations of PPC with AB (H6) and LE (H7). In Model 85, PPC, GM, and their interaction were included in each of the three regression equations. The LE equation additionally included AB, and the AA equation additionally included both AB and LE. H1 concerned the overall negative association between PPC and AA, examined using Pearson correlation analysis rather than the direct association conditional on the mediators. Bootstrap analysis with 5,000 resamples was used to generate 95% confidence intervals (CIs). An effect was considered significant at *α* = 0.05 if the interval excluded zero.

## 4 Results

### 4.1 Construct reliability and validity

The measurement properties were assessed via Cronbach’s α, composite reliability (CR), and average variance extracted (AVE). Standardized factor loadings ranged from 0.619 to 0.868, with item-level results reported in Supplementary Table S1. As shown in Table 2, Cronbach’s α ranged from 0.881 to 0.945, and CR ranged from 0.818 to 0.950, indicating adequate internal consistency. AVE values ranged from 0.517 to 0.568 and exceeded 0.50 for all constructs. The relatively lower AVE for AB (0.529) indicates limited evidence of convergent validity and warrants caution when interpreting results involving this construct.

**Table 2:** Reliability and validity.

Constructs	Cronbach’s *α*	CR	AVE
PPC	0.921	0.950	0.517
AB	0.881	0.818	0.529
LE	0.945	0.915	0.547
GM	0.888	0.905	0.547
AA	0.906	0.912	0.568

PPC, parental psychological control; AB, academic buoyancy; LE, learning engagement; GM, growth mindset; AA, academic adjustment.

The Heterotrait–Monotrait Ratio (HTMT) values ranged from 0.451 to 0.638, all below the recommended threshold of 0.90 (Henseler et al., 2014), indicating satisfactory discriminant validity among the constructs (see Table 3).

**Table 3:** HTMT of the constructs.

Construct	PPC	AB	LE	GM	AA
PPC	—				
AB	0.573	—			
LE	0.622	0.612	—		
GM	0.451	0.453	0.475	—	
AA	0.610	0.593	0.638	0.528	—

PPC, parental psychological control; AB, academic buoyancy; LE, learning engagement; GM, growth mindset; AA, academic adjustment.

### 4.2 Common method bias (CMB) and confirmatory factor analysis (CFA)

AMOS 26.0 was used to perform confirmatory factor analysis (CFA) to evaluate the structural validity of the measurement model and assess common method bias (CMB). The model showed good fit, and all indices met recommended cutoffs, indicating adequate structural validity. Possible CMB was further evaluated through the unmeasured latent method construct (ULMC) approach, in which an additional method factor was specified within the original model to absorb shared item variance attributable to measurement artifacts. As reported in Table 4, shifts in GFI, AGFI, CFI, IFI, and TLI were each below 0.01, and the change in RMSEA was below 0.05. Following Cheung and Rensvold (2002), suggest that incorporating the method factor did not significantly enhance model fit, indicating that substantial common method bias is unlikely in this study.

**Table 4:** Results of common method bias tests.

Model / Criterion	*χ*^2^(df)	*χ*^2^/df	RMSEA	SRMR	GFI	AGFI	CFI	IFI	TLI
Reference value		<5	<0.080	<0.080	>0.900	>0.900	>0.900	>0.900	>0.900
Baseline model	1140.635 (1024)	1.114	0.014	0.028	0.927	0.920	0.993	0.993	0.993
Method factor model	1090.471 (1023)	1.066	0.010	0.025	0.931	0.924	0.996	0.996	0.996
Model Fit Difference			∆RMSEA	∆SRMR	∆GFI	∆AGFI	∆CFI	∆IFI	∆TLI
			0.004	0.003	0.004	0.004	0.003	0.003	0.003
Evaluation criterion			<0.05	<0.05	<0.01	<0.01	<0.01	<0.01	<0.01

*χ*^2^, chi-square value; df, degree of freedom; RMSEA, root mean square error of approximation; SRMR, standardized root mean square residual; GFI, goodness-of-fit index; AGFI, adjusted goodness-of-fit index; CFI, comparative fit index; IFI, incremental fit index; TLI, Tucker-Lewis index.

### 4.3 Tolerance and collinearity diagnostics

Several indices can be applied to evaluate the structural model, including collinearity diagnostics, path coefficients, and the coefficient of determination. These indices help assess the model’s reliability and explanatory power. Collinearity diagnostics were conducted to determine whether multicollinearity existed among the variables (Leguina, 2015). Table 5 shows that all tolerance statistics exceeded 0.1 and all VIF values remained below 5, suggesting that serious multicollinearity was not evident. Therefore, multicollinearity was considered acceptable in this study.

**Table 5:** Collinearity diagnostics for the structural model.

Construct	Tolerance	VIF
PPC	0.579	1.727
AB	0.643	1.556
LE	0.566	1.765
GM	0.751	1.332

PPC, parental psychological control; AB, academic buoyancy; LE, learning engagement; GM, growth mindset.

### 4.4 Descriptive statistics

To verify that the data satisfied multivariate assumptions and to enhance result accuracy, the analysis assessed normality using skewness and kurtosis. Following Kim (2013), values with |Sk| < 2 and |Kur| < 7 indicate approximate normality. Table 6 indicates that each variable satisfied the required criteria.

**Table 6:** Descriptive statistics and correlations among the variables.

Construct	*M*	*SD*	Sk	Kur	PPC	AB	LE	AA	GM
PPC	49.39	14.930	0.248	−0.802	1				
AB	10.45	3.319	−0.005	−0.701	−0.514***	1			
LE	35.55	10.310	−0.149	−0.560	−0.585***	0.528***	1		
AA	35.36	9.488	−0.431	−0.566	−0.571***	0.511***	0.582***	1	
GM	30.71	8.033	−0.240	−0.588	−0.426***	0.389***	0.431***	0.479***	1

***p < 0.001. PPC, parental psychological control; AB, academic buoyancy; LE, learning engagement; GM, growth mindset; AA, academic adjustment.

Correlations were computed for PPC, AB, LE, AA, and GM. As reported in Table 6, PPC was negatively associated with AB (*r* = −0.514, *p* < 0.001), LE (*r* = −0.585, *p* < 0.001), AA (*r* = −0.571, *p* < 0.001), and GM (*r* = −0.426, *p* < 0.001). AB showed positive associations with LE (*r* = 0.528, *p* < 0.001), AA (*r* = 0.511, *p* < 0.001), and GM (*r* = 0.389, *p* < 0.001). LE was positively associated with AA (*r* = 0.582, *p* < 0.001) and GM (*r* = 0.431, *p* < 0.001). AA was also positively associated with GM (*r* = 0.479, *p* < 0.001). The results support H1.

### 4.5 Mediation analysis

With gender and grade controlled, Table 7 shows that PPC was significantly and negatively related to AB, LE, and AA. In addition, AB was significantly and positively related to both LE and AA, and LE was also positively related to AA. The bootstrap results are likewise reported in Table 8. The overall association between PPC and AA was −0.571. Of this, the direct component was −0.288, representing 50.43% of the total, whereas the indirect component was −0.283, accounting for 49.57%. These results indicate that the PPC-AA association involved both direct and indirect pathways. Specifically, the indirect effect through AB was −0.103 (18.09% of the total indirect effect), the indirect effect through LE was −0.131 (22.94%), and the indirect effect through AB and LE sequentially was −0.049 (8.56%). The results indicated significant statistical indirect associations involving AB, LE, and both variables in the specified serial model. These findings were consistent with H2, H3, and H4, but did not establish a temporal sequence or causal mediation.

**Table 7:** Association tests among variables in the chain mediation model.

Outcome variable	Predictor variable	*R*	*R^2^*	*F*	*β*	*SE*	*t*
AB	PPC	0.514	0.264	221.376***	−0.514	0.035	−14.879***
LE	PPC	0.642	0.412	216.176***	−0.426	0.036	−11.834***
	AB				0.309	0.036	8.593***
AA	PPC	0.668	0.447	165.637***	−0.288	0.039	−7.442***
	AB				0.201	0.037	5.440***
	LE				0.308	0.039	7.873***

***p < 0.001. PPC, parental psychological control; AB, academic buoyancy; LE, learning engagement; AA, academic adjustment.

**Table 8:** Bootstrap analysis of the serial mediation effects among parental psychological control, academic buoyancy, learning engagement, and academic adjustment.

Path type	Specific path	Effect size	Boot SE	Bootstrap 95% CI		%
				LLCI	ULCI	
Total effect	PPC → AA	−0.571	0.033	−0.636	−0.506	100.00
Direct effect	PPC → AA	−0.288	0.039	−0.364	−0.212	50.43
Total indirect effect	PPC → AA	−0.283	0.027	−0.339	−0.231	49.57
Indirect effect 1	PPC → AB → AA	−0.103	0.020	−0.142	−0.065	18.09
Indirect effect 2	PPC → LE → AA	−0.131	0.020	−0.173	−0.094	22.94
Indirect effect 3	PPC → AB → LE → AA	−0.049	0.010	−0.068	−0.032	8.56

PPC, parental psychological control; AB, academic buoyancy; LE, learning engagement; AA, academic adjustment.

### 4.6 Moderation analysis

Building on the serial mediation framework, GM was incorporated as a moderator using PROCESS Model 85. As shown in Table 9, the PPC × GM interaction was significant and positive in the regression equations for AB (*β* = 0.136, *p* < 0.001), LE (*β* = 0.118, *p* = 0.001), and AA (*β* = 0.129, *p* < 0.001). These results indicated that GM moderated the associations of PPC with AB and LE, as well as its direct association with AA after accounting for both mediators, supporting H5–H7.

**Table 9:** Tests of the moderated serial mediation model.

Outcome	Predictor	*β*	*t*	95% CI	*R^2^*	*F*
AB	PPC	−0.398	−10.603	[−0.471, −0.324]	0.316	94.736
	GM	0.189	5.078	[0.116, 0.262]		
	PPC × GM	0.136	3.873	[0.067, 0.205]		
LE	PPC	−0.360	−9.790	[−0.432, −0.288]	0.446	123.672
	AB	0.250	6.893	[0.179, 0.321]		
	GM	0.154	4.508	[0.087, 0.221]		
	PPC × GM	0.118	3.686	[0.055, 0.181]		
AA	PPC	−0.239	−6.296	[−0.313, −0.164]	0.489	117.619
	AB	0.153	4.235	[0.082, 0.224]		
	LE	0.241	6.228	[0.165, 0.317]		
	GM	0.185	5.545	[0.120, 0.251]		
	PPC × GM	0.129	4.154	[0.068, 0.191]		

PPC, parental psychological control; AB, academic buoyancy; LE, learning engagement; GM, growth mindset; AA, academic adjustment.

Simple slope analyses further described the moderation (see Figures 2–4). At low GM (M − 1 SD), PPC was negatively associated with AB (*β* = −0.535, SE = 0.047, *p* < 0.001). At high GM (M + 1 SD), the association was less negative (*β* = −0.261, SE = 0.049, *p* < 0.001). Thus, the negative association between PPC and AB was weaker at higher GM.

**Figure 2:**
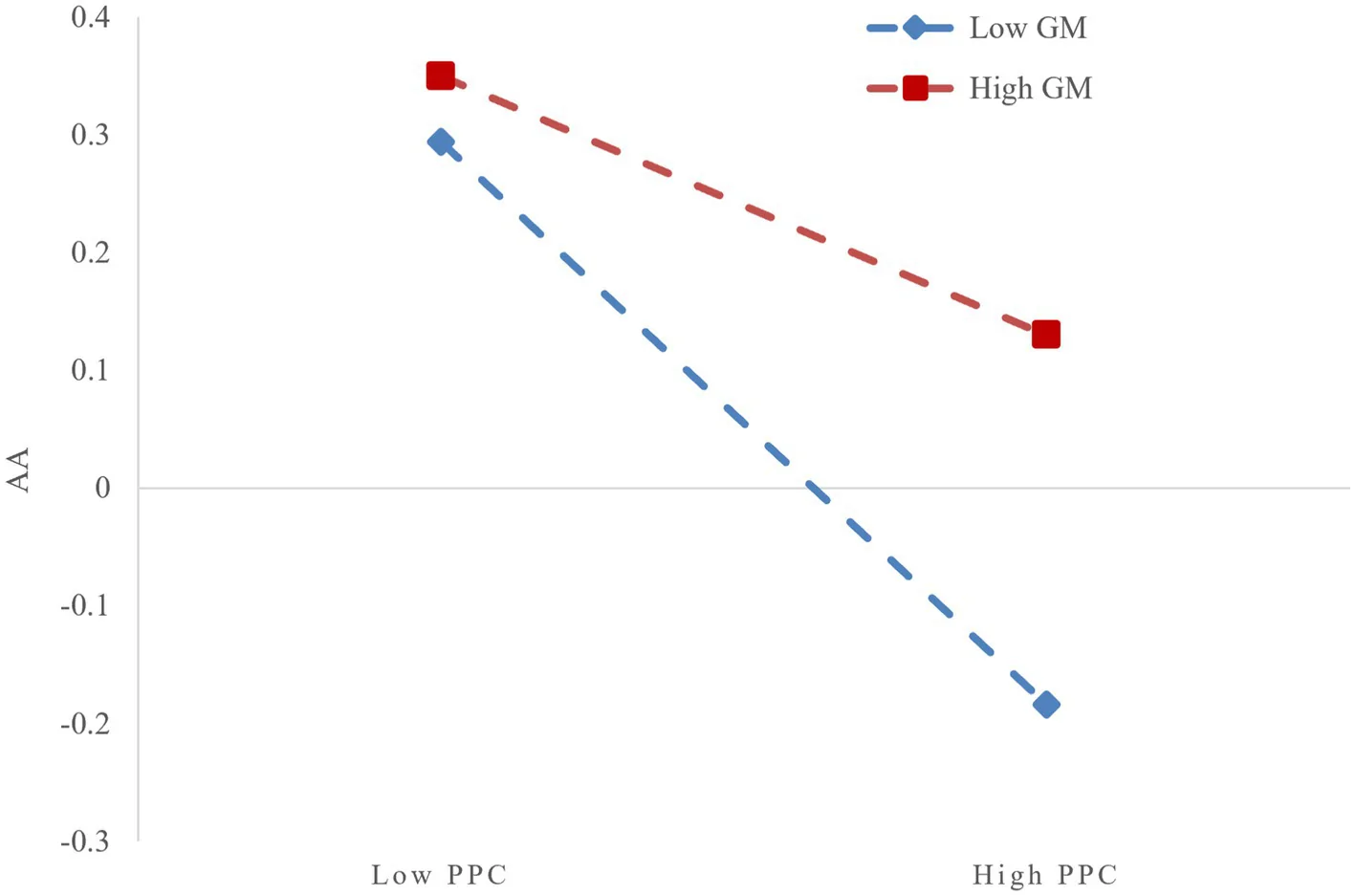
The moderating role of growth mindset in the relationship between parental psychological control and academic adjustment.

**Figure 3:**
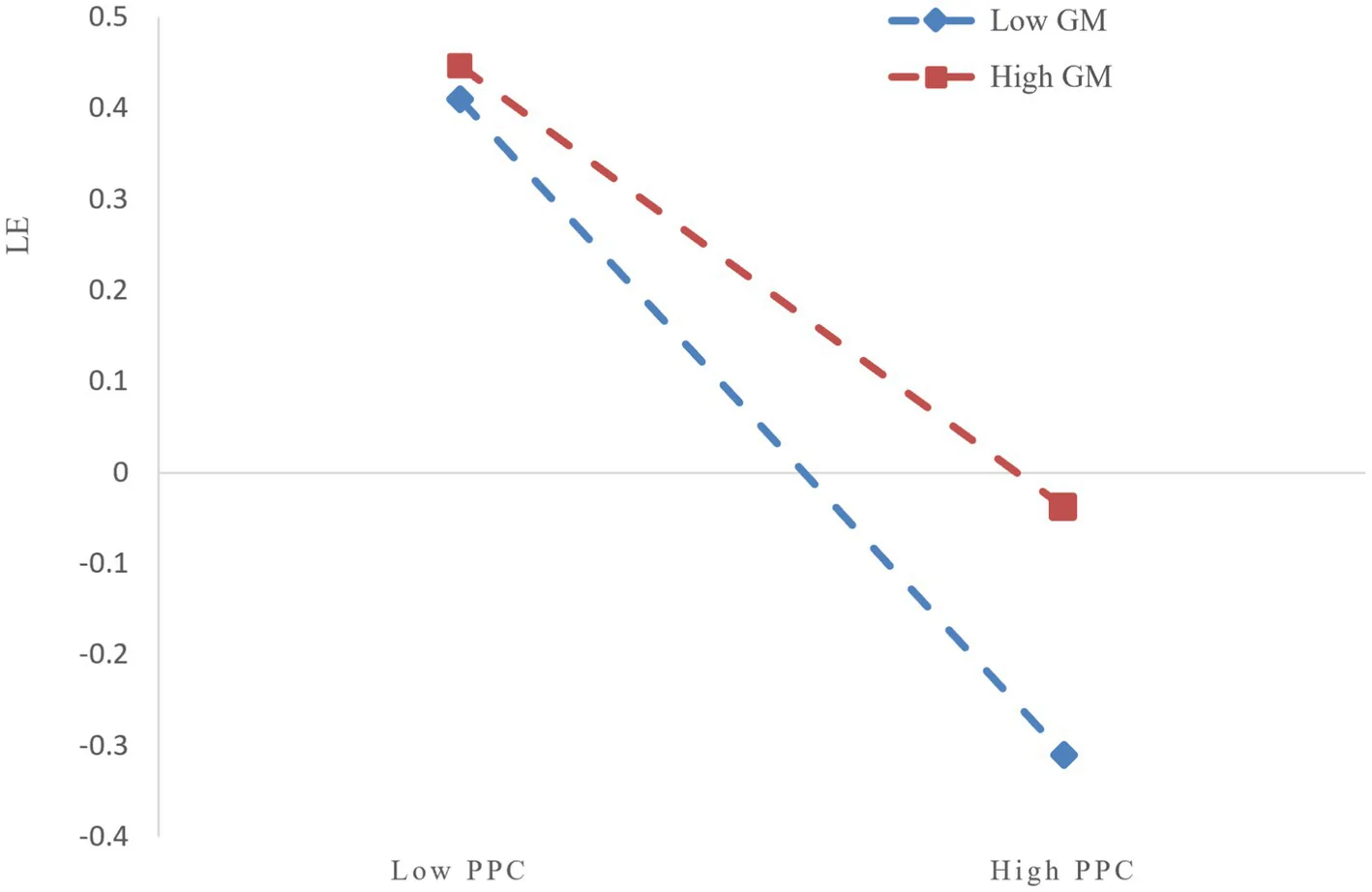
The moderating role of growth mindset in the relationship between parental psychological control and learning engagement.

**Figure 4:**
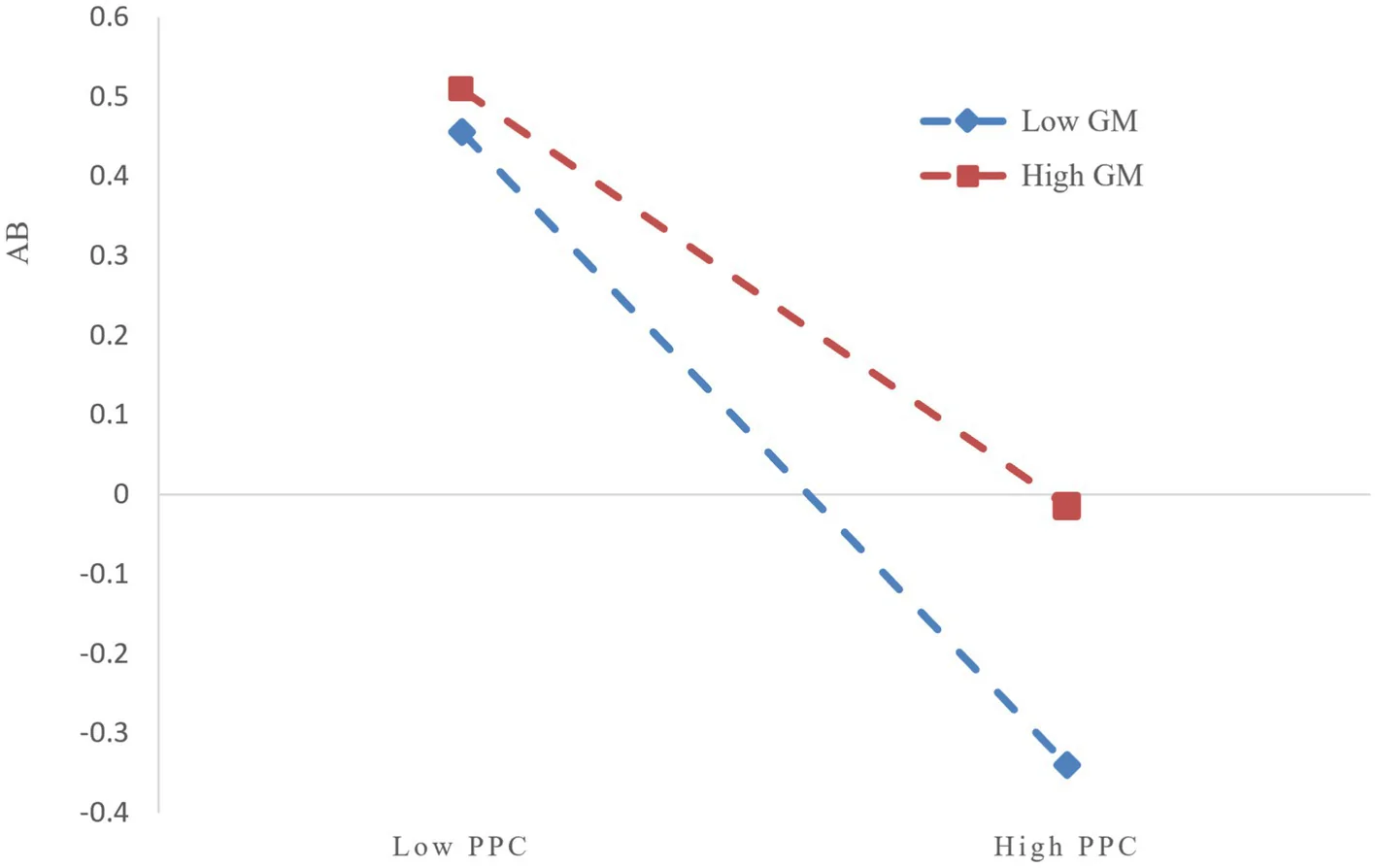
The moderating role of growth mindset in the relationship between parental psychological control and academic buoyancy.

For LE, the association with PPC, after accounting for AB, was negative at low GM (*β* = −0.478, SE = 0.045, *p* < 0.001) and less negative at high GM (*β* = −0.242, SE = 0.050, *p* < 0.001). Thus, the negative association between PPC and LE was weaker at higher GM. Regarding AA, the direct association with PPC, after accounting for AB and LE, was negative at low GM (*β* = −0.368, SE = 0.041, *p* < 0.001) and less negative at high GM (*β* = −0.110, SE = 0.045, *p* = 0.015). Thus, the negative direct association between PPC and AA was weaker at higher GM.

As shown in Table 10, the conditional indirect associations between PPC and AA through AB, LE, and the sequential pathway involving AB and LE were negative at low, mean, and high levels of GM, with all 95% bootstrap confidence intervals excluding zero. These negative indirect associations became weaker as GM increased. Furthermore, all three indices of moderated mediation were positive, and their 95% bootstrap confidence intervals excluded zero, supporting the moderating role of GM in the indirect associations through AB, LE, and the sequential AB-to-LE pathway.

**Table 10:** Conditional indirect effects and indices of moderated mediation.

Indirect association via	GM	Estimate	95% bootstrap CI	Index	95% CI (index)
AB	Low	−0.084	[−0.127, −0.045]	0.021	[0.009, 0.037]
	Mean	−0.060	[−0.091, −0.032]		
	High	−0.039	[−0.066, −0.017]		
LE	Low	−0.118	[−0.163, −0.077]	0.029	[0.013, 0.048]
	Mean	−0.086	[−0.122, −0.055]		
	High	−0.057	[−0.094, −0.029]		
AB → LE	Low	−0.033	[−0.050, −0.019]	0.008	[0.004, 0.014]
	Mean	−0.024	[−0.037, −0.013]		
	High	−0.016	[−0.028, −0.007]		

AB, academic buoyancy; LE, learning engagement; GM, growth mindset.

## 5 Discussion

The results showed a negative association between PPC and adolescents’ AA, supporting H1. Higher PPC aligned with lower AA. This pattern accords with Cui et al. (2014), who reported a negative link between PPC and adolescents’ adjustment. PPC restricts autonomy, weakens perceived competence, and undermines emotional security, which together deplete the motivation and psychological resources needed to handle academic demands (Xu et al., 2024). Adolescents who report greater PPC may also view learning as an externally assigned duty rather than a self-directed activity. Such motivational experiences offer a possible theoretical explanation for the negative association between PPC and AA (Cui et al., 2014), although the present study did not directly assess these processes. These findings suggest that parenting practices warrant attention in research on adolescents’ academic functioning. Whether reducing psychological control or strengthening need-supportive parenting improves AA requires examination in intervention studies.

A significant statistical indirect association between PPC and adolescents’ AA involved AB, consistent with H2. Higher PPC was associated with lower AB, and lower AB was associated with poorer AA. From an SDT perspective, basic psychological need satisfaction provides a possible theoretical explanation for the association between PPC and AB (Nogg et al., 2021). Lower AB may also be associated with greater difficulty managing academic stress and poorer AA (Safriani and Muhid, 2022). The mediation observed here reflects the link between psychological need satisfaction and AA. By undermining environments that satisfy basic psychological needs, PPC hampers the development of AB and interrupts the pathway to effective AA. This mechanism clarifies how family context relates to school functioning and points to AB as a promising target for intervention.

PPC showed a significant statistical indirect association with adolescents’ AA involving LE, consistent with H3. SDT posits that sustained LE rests on the fulfillment of autonomy, competence, and relatedness (Nagpaul and Chen, 2019). Lower need satisfaction may provide a theoretical explanation for the association between PPC and LE. Students who report greater PPC may also report lower engagement and poorer AA. Manipulative and intrusive practices, including emotional coercion and overinvolvement, may be associated with lower vitality, focus, and persistence during learning (Zhang and Peng, 2025). Lower engagement is associated with poorer AA (García-Ros et al., 2018). The observed indirect association is consistent with LE being relevant to the relationship between family context and AA, but does not establish LE as a causal intermediary or directly test the role of psychological need satisfaction.

A significant statistical indirect association involving AB and LE in the specified serial model was consistent with H4. The proposed theoretical account concerns students’ perceptions of autonomy, competence, and relatedness, as well as beliefs about their abilities (Dweck, 2013). These considerations motivated the hypothesized association between PPC and AB. Lower AB was also expected to be associated with lower LE (Ershadi Chahardeh, 2020). However, the present findings do not establish that PPC precedes lower AB or that lower AB precedes lower LE. The specified ordering describes a statistical pattern of associations among PPC, AB, LE, and AA. This pattern suggests hypotheses for future longitudinal research, while the effects of interventions involving AB or LE require separate evaluation.

The moderation analyses indicated that the associations of PPC with AA, AB, and LE varied across levels of GM, consistent with H5, H6, and H7. The negative direct association between PPC and AA, after accounting for AB and LE, was weaker at higher GM. This result accords with Yeager and Dweck (2020), who argued that a GM coaches people to reframe challenges as avenues for growth, not threats to self-esteem. One possible explanation is that adolescents with higher GM appraise psychologically controlling parenting as a manageable external condition rather than a denial of personal competence. Such appraisals may be associated with greater perceived autonomy and competence and better AA (Wang et al., 2024), although this explanation was not directly tested in the present study. The conditional indirect associations involving AB and LE also varied across levels of GM. For the association involving AB, one possible explanation is that beliefs about the malleability of ability are associated with appraising difficulties as temporary and manageable. Such beliefs may be associated with greater AB and more adaptive responses to academic difficulties (Liu, 2025). However, the present data do not establish that GM preserves AB under psychological control or that changes in AB explain changes in AA. For the association involving LE, adolescents with higher GM may view learning as an opportunity for capability development rather than a test of fixed intelligence (Chen et al., 2024). This orientation offers a possible explanation for the weaker negative association between PPC and LE at higher GM. The present findings do not establish that GM sustains intrinsic interest or engagement over time, or that these processes account for the association with AA. Taken together, the results indicate that the negative direct and indirect associations between PPC and AA were weaker at higher GM. These findings describe variation in the observed associations, but do not demonstrate a protective causal effect of GM. Future intervention studies could examine whether combining GM training with family support improves adolescents’ AA.

## 6 Implications

### 6.1 Theoretical implications

First, this study proposed a moderated serial mediation framework for adolescents’ AA grounded in SDT. The model estimates direct and indirect associations between PPC and AA involving AB and LE, and examines their variation across levels of GM. The findings are compatible with the proposed SDT-informed account, but do not directly test psychological need satisfaction or establish the causal processes proposed by the theory.

Second, the research identifies statistical indirect associations involving AB and LE. Higher PPC was associated with lower AB, lower LE, and poorer AA, and the specified serial indirect association was significant. These findings describe a pattern consistent with the proposed model and suggest hypotheses for future research. Whether AB precedes LE and whether changes in these variables contribute to changes in AA require longitudinal and intervention evidence.

Finally, the study examined GM as a moderator in this model. GM moderated the association between PPC and AA and moderated the indirect associations through AB and LE. This specification describes how the associations among the study variables vary across levels of GM. It may inform hypotheses about differences among adolescents and guide future evaluations of growth-mindset–oriented educational interventions.

### 6.2 Practical implications

First, the findings indicate that PPC is negatively associated with adolescents’ AA. This association suggests that family interactions warrant consideration in academic support research. Parental education and need-supportive family practices are possible areas for intervention evaluation, but the present study does not establish that these approaches improve AA.

Second, the statistical indirect associations involving AB and LE suggest that both variables may be relevant to research on adolescents’ AA. Future studies could evaluate coordinated support involving responses to academic setbacks, participation in learning tasks, and need-supportive parenting. Controlled evaluations are needed to determine whether such approaches improve AA and whether any changes involve AB or LE.

Finally, the variation in associations across levels of GM suggests that GM may be relevant when designing and evaluating academic support. Future studies could test whether students with different GM levels respond differently to challenging learning tasks, metacognitive training, or feedback concerning the malleability of ability. The present results do not establish the effectiveness of these approaches or justify assigning specific interventions on the basis of GM alone.

## 7 Limitations and future directions

This study examined associations between PPC and adolescents’ AA, but several limitations remain. First, the sample comprised only adolescents, which constrains representativeness and the generalizability of conclusions. Future work can include other developmental stages, such as college freshmen, to examine whether these associations differ across groups. Second, the cross-sectional design limits causal inference among variables. Longitudinal tracking or controlled interventions can more rigorously examine the causal pathways linking PPC and AA. Third, self-reporting alone introduces social desirability and subjective biases. Subsequent studies can integrate multi-informant ratings, behavioral observations, and physiological indicators to strengthen reliability and ecological validity. Fourth, AA was assessed through investment in school, which captures the importance students attach to academic success. The findings should therefore be interpreted within this specific domain. Future research should include broader measures of academic adjustment, such as adaptation to academic demands and academic performance.

## 8 Conclusion

Based on SDT, a moderated serial mediation framework was constructed in this study, and questionnaire data from 622 adolescents were used to explore how PPC was associated with AA. Results showed a negative association between PPC and AA. AB and LE each mediated this association, and their serial pathway also played a role. GM moderated the association between PPC and AA, and it moderated the indirect pathways through AB and LE. These findings illuminate psychological processes underlying adolescents’ AA and offer empirical support for the application of SDT in educational contexts. Given the single-population sample and self-reported data, future research can broaden educational stages and employ objective behavioral indicators or multi-wave designs to enhance external validity and strengthen causal interpretation.

## Data Availability

The raw data supporting the conclusions of this article will be made available by the authors, without undue reservation.
