# Supplementary material for: Parental psychological control and adolescents’ academic adjustment: a moderated serial mediation model
Source: Front Psychol. 2026 Oct 19;17:1910535. doi: 10.3389/fpsyg.2026.1910535 (PMC13724605; doi:10.3389/fpsyg.2026.1910535)
Supplement: Supplementary file 1 [file Table_1.DOCX]

**Supplementary Material**

**Manuscript title:**

Parental Psychological Control and Adolescents’ Academic Adjustment: A Moderated Serial Mediation Model

Table S1. Standardized Factor Loadings of Measurement Items

| Construct | Item | Loading |
| --- | --- | --- |
| PPC | PPC1 | 0.756 |
|  | PPC2 | 0.739 |
|  | PPC3 | 0.752 |
|  | PPC4 | 0.752 |
|  | PPC5 | 0.733 |
|  | PPC6 | 0.740 |
|  | PPC7 | 0.745 |
|  | PPC8 | 0.760 |
|  | PPC9 | 0.738 |
|  | PPC10 | 0.754 |
|  | PPC11 | 0.757 |
|  | PPC12 | 0.754 |
|  | PPC13 | 0.655 |
|  | PPC14 | 0.649 |
|  | PPC15 | 0.657 |
|  | PPC16 | 0.657 |
|  | PPC17 | 0.619 |
|  | PPC18 | 0.693 |
| AB | AB1 | 0.725 |
|  | AB2 | 0.671 |
|  | AB3 | 0.772 |
|  | AB4 | 0.739 |
| LE | LE1 | 0.635 |
|  | LE2 | 0.691 |
|  | LE3 | 0.728 |
|  | LE4 | 0.806 |
|  | LE5 | 0.819 |
|  | LE6 | 0.754 |
|  | LE7 | 0.705 |
|  | LE8 | 0.650 |
|  | LE9 | 0.837 |
| AA | AA1 | 0.627 |
|  | AA2 | 0.628 |
|  | AA3 | 0.729 |
|  | AA4 | 0.815 |
|  | AA5 | 0.868 |
|  | AA6 | 0.806 |
|  | AA7 | 0.664 |
|  | AA8 | 0.837 |
| GM | GM1 | 0.656 |
|  | GM2 | 0.703 |
|  | GM3 | 0.753 |
|  | GM4 | 0.793 |
|  | GM5 | 0.825 |
|  | GM6 | 0.805 |
|  | GM7 | 0.702 |
|  | GM8 | 0.657 |

*Note: PPC: Parental Psychological Control; AB: Academic Buoyancy; LE: Learning Engagement; GM: Growth Mindset; AA: Academic Adjustment.*
